# Impact of Adalimumab Treatment on Interleukin-17 and Interleukin-17 Receptor Expression in Skin and Synovium of Psoriatic Arthritis Patients with Mild Psoriasis

**DOI:** 10.3390/biomedicines10020324

**Published:** 2022-01-29

**Authors:** Janne W. Bolt, Arno W. van Kuijk, Marcel B. M. Teunissen, Dennis van der Coelen, Saïda Aarrass, Daniëlle M. Gerlag, Paul P. Tak, Marleen G. van de Sande, Maria C. Lebre, Lisa G. M. van Baarsen

**Affiliations:** 1 Department of Rheumatology and Clinical Immunology, Amsterdam Rheumatology & Immunology Center (ARC), Amsterdam UMC, University of Amsterdam, 1105 AZ Amsterdam, The Netherlands; j.w.bolt@amsterdamumc.nl (J.W.B.); kir@amsterdamumc.nl (D.v.d.C.); kir@amc.uva.nl (S.A.); dmgerlag@gmail.com (D.M.G.); tak.paulpeter@gmail.com (P.P.T.); m.g.vandesande@amsterdamumc.nl (M.G.v.d.S.); 2 Department of Rheumatology, Amsterdam Rheumatology & Immunology Center (ARC)-Reade, 1040 HG Amsterdam, The Netherlands; a.v.kuijk@reade.nl; 3 Department of Rheumatology, Reade, 1056 AB Amsterdam, The Netherlands; 4 Department of Dermatology, Amsterdam UMC, University of Amsterdam, 1105 AZ Amsterdam, The Netherlands; m.b.teunissen@amsterdamumc.nl; 5 Candel Therapeutics, Needham, MA 02494, USA; 6 Internal Medicine, Cambridge University, Cambridge CB2 1TN, UK; 7 The Netherlands Cancer Institute, Division of Pharmacology, Plesmanlaan 121, 1066 CX Amsterdam, The Netherlands; c.lebre@nki.nl

**Keywords:** psoriatic arthritis, skin, synovium, interleukin-17, adalimumab, immunopathogenesis, immunohistochemistry

## Abstract

Interleukin (IL)-17 and tumor necrosis factor-alpha (TNF)-α are key players in psoriatic arthritis (PsA) pathogenesis. While both cytokines can be therapeutically targeted with beneficial clinical outcome, it is unclear whether inhibiting one cytokine will affect the other at sites of inflammation. If both act independently, this might provide a rationale for dual or combined inhibition of both cytokines. Here, we evaluated the effect of TNF blockade in PsA patients on IL-17 levels in both skin and synovial tissue biopsies. PsA patients with mild psoriatic skin lesions were randomized to receive either adalimumab or placebo for four weeks. Synovial and skin biopsies were obtained at weeks zero and four. Skin from healthy donors (HDs) was used for comparison. Expression of IL-17A, IL-17F, IL-17RA and IL-17RC was assessed by immunohistochemistry and analyzed with digital image analysis. We found relatively low levels of IL-17 and its receptors in the skin of PsA patients compared to HD, and only IL-17F in the dermis of lesional psoriatic skin was significantly higher compared to HD skin (*p* = 0.0002). Histologically IL-17A, IL-17F, IL-17RA and IL-17RC in skin and synovial tissue were not downregulated by adalimumab treatment. Thus, in this cohort of PsA patients with mild psoriasis, TNF blockade did not affect the protein levels of IL-17 cytokines and its receptors in skin and synovium, despite reduced cellular inflammation and improved clinical outcome for joint involvement.

## 1. Introduction

Psoriatic arthritis (PsA) is a chronic inflammatory joint disorder characterized by musculoskeletal manifestations such as arthritis, spondylitis, dactylitis spinal involvement and enthesitis, and extra articular features such as psoriatic skin lesions [1]. The use of conventional synthetic disease modifying antirheumatic drugs (csDMARDs), and especially biological (b) DMARDs, has drastically improved treatment outcomes in PsA. Despite this, disease remission is still not achieved in all PsA patients, and the efficacy of the various treatments differs from one person to the next, and can decrease over time [2]. The pathogenesis of PsA is not yet fully elucidated, but it is clear that the immune pathways active in PsA can vary according to the site of tissue inflammation. Tumor necrosis factor-alpha (TNF-α) and interleukin (IL)-17 are key cytokines in the pathogenesis of PsA [1]. IL-17A is the most widely studied and most biological active cytokine of the IL-17 family (IL17 A-F), and is produced by, e.g., CD4+ T cells, CD8+ T cells, gamma delta T cells, natural killer T cells (NKT), mast cells, and innate lymphoid cells [3,4,5]. Gene expression and protein levels of IL-17A, C and F are increased in affected skin lesions, nonlesional skin and synovial tissue of PsA patients [3,6], which result in the release of other pro-inflammatory cytokines and chemokines, the promotion of angiogenesis, and bone remodeling [7]. IL-17A works in synergy with IL-17F; these cytokines are similar, although the latter is less potent than the former [4]. Little is known about the function of IL-17C and the other IL-17 family members (IL-17D and IL-17E) in PsA [8].

TNF-α is upregulated in the blood, skin and synovium of PsA patients [9,10], and is mainly produced by macrophages. By binding to TNF receptors, TNF-α activates kinases, phosphor-proteins and nuclear factor kappa B, which leads to pro-inflammatory gene transcription, cytokine secretion, cytotoxic effects and differentiation of T-helper cells [11]. In psoriatic skin, TNF-α can activate myeloid dendritic cells (DCs) that produce IL-23, resulting in the activation of IL-17A secreting T cells and innate lymphoid cells [12,13]. Additionally, it was shown in synovium of rheumatoid arthritis patients that TNF-α stimulates attraction of DCs and IL-17A secreting T cells [14]. The importance of the TNF-α and IL-23/IL-17A axis in disease pathogenesis is well described for psoriasis [15] and psoriatic arthritis [16].

TNF-α also synergizes with IL-17 to stimulate inflammation in general, and studies in rheumatoid arthritis patients, in both ex vivo and gene expression experiments, showed that this results in increased levels of inflammatory mediators, higher levels of granulopoiesis and exacerbation of bone destruction by higher production of RANKL [17,18].

Both TNF inhibitors and IL-17A inhibitors are effective in the treatment of PsA [19,20,21,22,23]. Therapeutic application of bDMARDS, including TNF inhibitors, IL-17A inhibitors, or IL-12/IL-23 inhibitors, is advised after failure of csDMARDs treatment [24,25,26]. Compared to TNF inhibitors, IL-17A inhibitors have shown superior therapeutic efficacy on psoriatic skin lesions in PsA, and may therefore be primarily considered in patients with significant skin involvement [17,27,28]. As there is still no ideal treatment for PsA, i.e., one that induces remission in all patients, it is important to determine whether IL-17 expression is affected by the inhibition of TNF-α, and vice versa, or whether both bDMARDS downregulate inflammation by independent mechanisms. TNF blockers, such as adalimumab, might also affect the synergistic effects between IL-17 and TNF, and as a consequence, diminish the inflammatory process without directly affecting IL-17. Currently, information is lacking on whether TNF blockade is partly effective due to indirect modulation of the levels of IL-17A, IL-17F and their receptors in (nonlesional and lesional) the skin and synovium of PsA patients.

The purpose of this exploratory study was to investigate the impact of TNF blockade on IL-17 levels in target tissues by evaluating the effects of adalimumab treatment on IL-17A, IL-17F and their receptors in paired skin and synovial tissue biopsies of patients suffering from PsA with moderate psoriasis lesions.

## 2. Materials and Methods

### 2.1. Study Design

As described in the original study [29,30], we performed a randomized, double-blind, placebo-controlled, single-center study at the Amsterdam UMC of the University of Amsterdam (Current Controlled Trials ISRCTN23328456). The local Medical Ethics Committee approved the study, and patients gave their written informed consent before they participated in the study. We collected information from the medical history of the study patients, and patients underwent physical examination by both a rheumatologist and a dermatologist. After randomization, patients received either adalimumab (*n* = 12) or placebo (*n* = 12) at baseline (day 1) and day 15 as previously described [29,30]. Twenty-four PsA patients that fulfilled the ClASsification of Psoriatic ARthritis (CASPAR) criteria aged 18–80 years were included in the study [31,32]. Patients were eligible if they had at least two tender and two swollen joints out of the 68 joints assessed for tenderness and 66 joints assessed for swelling. One of the swollen joints had to be accessible for arthroscopy. Concomitant treatment with nonsteroidal anti-inflammatory drugs (NSAIDs) and/or methotrexate was allowed if the dosage was stable for a minimum of 28 days. Other DMARDs were not allowed 1 month prior baseline, and a washout period of 2 months was necessary for leflunomide use. Parenteral, intra-articular or oral use of corticosteroids within 28 days before enrolment into the study was not allowed. Topical treatments for psoriasis were not allowed 14 days prior to baseline, except for low potency (class I) topical steroids to be used on scalp, palms, groin and/or soles of feet only [29,30]. Demographics and clinical features at baseline from the original study can be found in Table 1 [29].

The mean psoriasis area severity index (PASI) did not significantly change in the placebo group and the adalimumab group [30]. The mean disease activity score (DAS) 28 decreased significantly from 4.67 (SD 0.98) to 2.87 (1.27) in the adalimumab group, while the DAS28 remained approximately stable in the placebo group [29].

### 2.2. Skin and Synovial Tissue Biopsies

At baseline and week 4, patients had to undergo 4-mm punch biopsies from the lesional and nonlesional skin, preferentially from areas that were not exposed to sun. The second biopsy was preferably taken from the same psoriatic plaque that was targeted at baseline, separated by at least 1 cm [30]. We collected skin samples from 22 PsA patients. Available tissue data from baseline lesional and nonlesional PsA skin samples were compared with normal adult skin (*n* = 15) obtained from healthy donors (HD)s undergoing plastic surgery of the breast or abdomen. A mini-arthroscopy of an actively inflamed joint (knee, ankle, or wrist) was performed under local anesthesia in all patients before treatment and 28 days (same joint) after initiation of study medication [29]. 

Paired skin samples from 18 patients and paired synovial samples from 19 PsA patients were available for analysis [29,30]. 

The tissue samples were randomly coded and after this snap-frozen in Tissue-Tek OCT compound (Sakura Finetek Europe, Zoeterwoude, The Netherlands) by immersion in liquid nitrogen. Before immunohistochemical stainings, five-micrometer cryostat sections were cut and mounted on glass slides and stored at −80 °C.

### 2.3. Skin and Synovial Biopsy Immunohistochemical Staining

Skin and synovial tissue were stained with mouse monoclonal antibodies against IL-17A (IgG1, clone 41802), IL-17F (IgG2a, clone 197315) and their receptors IL-17 receptor(R)A (IgG1, clone 133617) and IL-17RC (IgG2b, clone 309882), all from R&D Systems (Minneapolis, MN, USA). Briefly, after tissue fixation with acetone, endogenous peroxidase activity was inhibited using 0.1% sodium azide and 0.3% hydrogen peroxide in phosphate buffered saline (PBS). The primary mouse monoclonal antibodies against human IL-17A, IL-17F and their receptors IL-17RA and IL-17RC were incubated overnight at 4 °C followed by secondary antibody affinity-purified horseradish peroxidase (HRP)-conjugated goat antimouse (Dako Cytomation, Glostrup, Denmark) for 30 min. After this step incubation with biotinylated tyramide (Perkin Elmer, Boston, MA, USA) was performed for 30 min and HRP-conjugated streptavidin was performed for 30 min (to detect IL-17A, IL-17F and IL-17RA) or followed by BrightVision (Immunologic, Duiven, The Netherlands; to detect IL-17RC). Detection of HRP-activity was performed with of hydrogen peroxide as substrate and amino ethylcarbazole (AEC; SK-4200; Vector Laboratories, Burlingame, CA, USA) as dye resulting in positive cells staining in red. Mayer’s hematoxilin (Merck, Darmstadt, Germany) was used for counterstaining and, after washing with distilled water, mounted in Kayser’s glycerol gelatine (Merck). As negative control, irrelevant isotype-matched immunoglobulins were applied to the sections instead of the primary antibody.

### 2.4. Quantification of IL-17A-, IL-17F-, IL17RA- and IL-17RC-Expressing Cells in Skin and Synovium

Quantification of IL-17A-, IL-17F-, IL17RA- and IL-17RC-expressing cells in PsA skin and synovial tissues was performed by computer-assisted image analysis, as previously described [29]. Briefly, after immunohistochemical staining, all coded sections (one section per patient per time-point) were randomly analyzed (18 high-power fields from different parts of the section were analyzed; the mean of the 18 high-power fields was calculated). The images of the high-power fields were analyzed using the Qwin analysis system (Leica, Cambridge, UK). The expression of stained proteins was calculated for each section as the median integrated optical density (IOD)/mm^2^ [33].

### 2.5. Statistical Analysis

The expression of IL-17A, IL-17F, IL17RA and IL-17RC in HD skin and PsA at baseline were compared using GraphPad Prism Software (V.5, GraphPad Software, La Jolla, CA, USA) and evaluation of statistical differences were performed with Kruskall-Wallis test with post-Dunn’s multiple comparison tests or nonparametric Mann Whitney U-test where appropriate. We considered *p* values below 0.05 as statistically significant. SPSS version 15.0 for Windows (SPSS, Chicago, IL, USA) was used to evaluate statistical differences between the effects of each treatment group (placebo vs. adalimumab). An analysis of covariance model (ANCOVA) after rank transformation was used to correct for baseline differences [34].

## 3. Results

### 3.1. Expression of IL-17A, IL-17F and Their Receptors in Skin of Psoriatic Arthritis Patients Compared to Healthy Donors

IL-17 levels were analyzed in the skin of 15 HDs and compared with lesional and nonlesional skin samples from 22 PsA patients.

**Figure 1:**
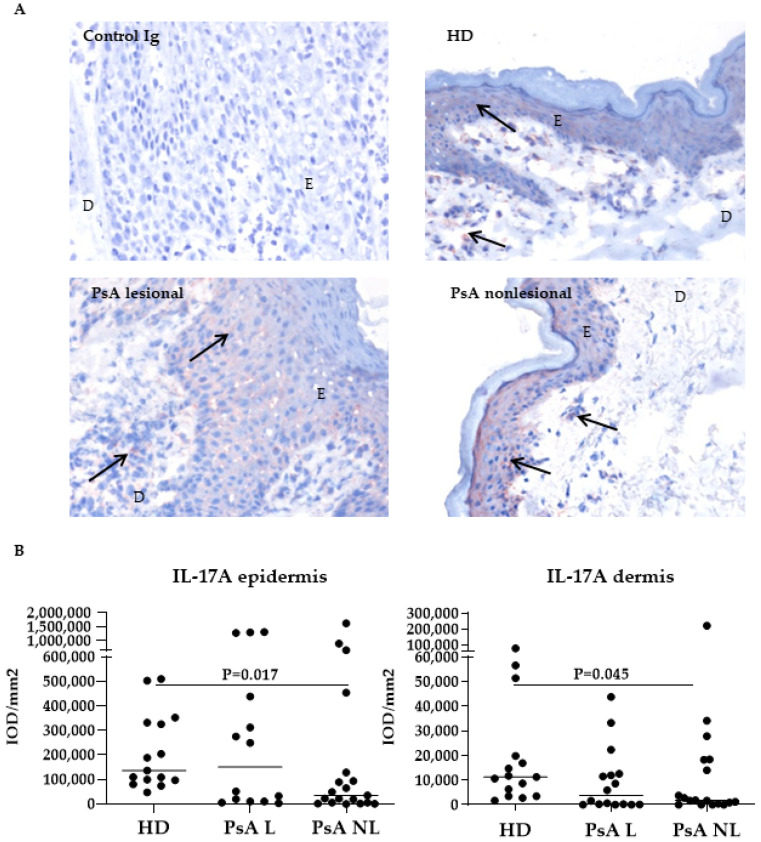
Expression of IL-17A was lower in the epidermis and dermis of nonlesional psoriatic skin compared to healthy donor skin. (**A**) Representative immunohistochemical staining of baseline IL-17A expression (arrows) in skin from HD and PsA patients. Original magnification 200 ×. (**B**) Quantification of IL-17A (Kruskal-Wallis test, epidermis *p* = 0.07, dermis *p* = 0.07) and expression in HD, PsA L skin and PsA NL skin. Results are shown as median IOD/mm^2^ and IQR of epidermis and dermis of HD and PsA patients. Per subject the median expression level of 18 high-power fields is used for analysis. E, epi-dermis; D, dermis; IL, interleukin; HD, healthy donor; PsA, psoriatic arthritis; L; lesional, NL; nonlesional, IOD; integrated optical density, IQR; interquartile range.

**Figure 2:**
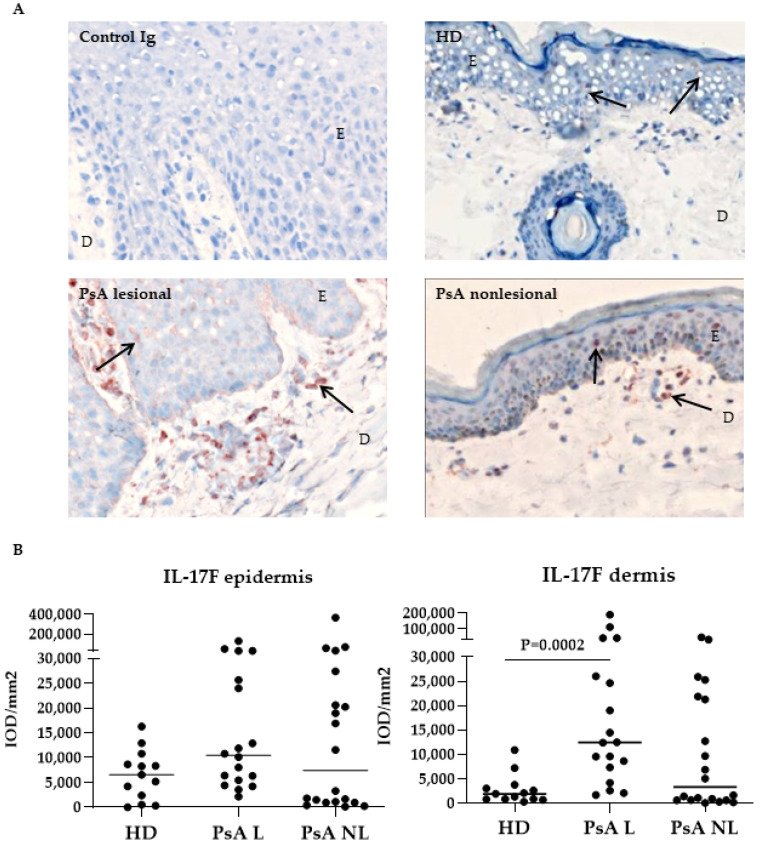
Expression of IL-17F was higher in the dermis of lesional psoriatic skin compared to healthy donor skin. (**A**) Representative immunohistochemical staining of baseline IL-17F expression (arrows) in skin from HD and PsA patients. Original magnification 200 ×. (**B**) Quantification of IL-17F (Kruskal-Wallis test, epidermis *p* = 0.17, dermis *p* = 0.0002) expression in HD, PsA L skin and PsA NL skin. Results are shown as median IOD/mm^2^ and IQR of epidermis and dermis of HD and PsA patients. Per subject the median expression level of 18 high-power fields is used for analysis. *p* values given in the graphs are cal-culated from a post Dunn’s test. E, epidermis; D, dermis; IL, interleukin; HD, healthy donor; PsA, psoriatic arthritis; L; lesional, NL; nonlesional, IOD; integrated optical density, IQR; interquartile range.

**Figure 3:**
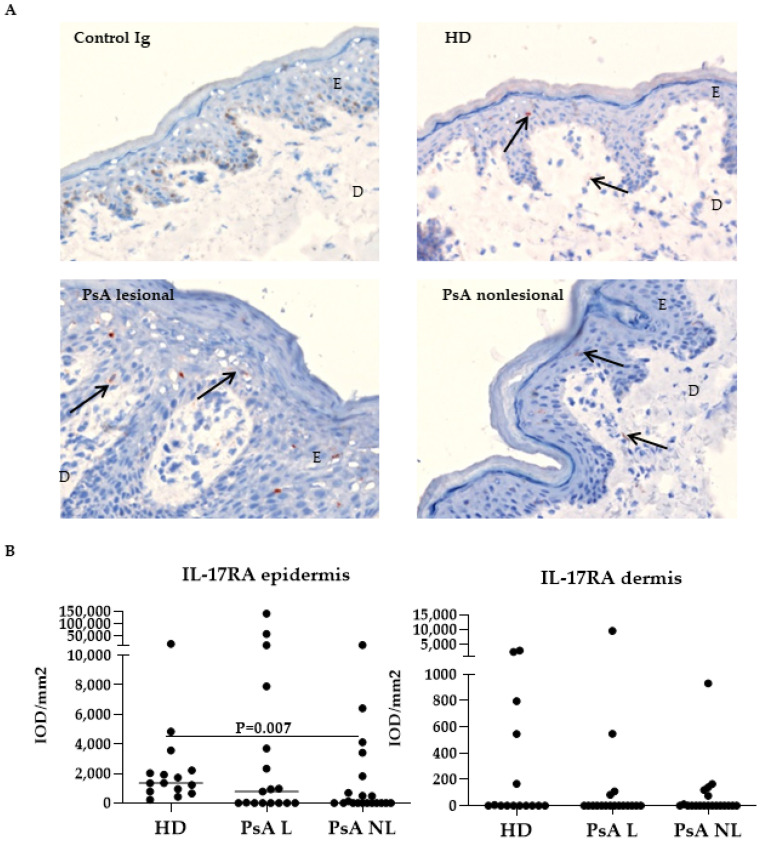
Expression of IL-17RA was lower in the epidermis of the nonlesional psoriatic skin compared to healthy donor skin. (**A**) Representative immunohistochemical staining of baseline IL-17RA expression (arrows) in skin from HD and PsA patients. Original magnification 200 ×. (**B**) Quantification of IL-17RA (Kruskal-Wallis test, epidermis *p* = 0.03, dermis *p* = 0.007) expression in HD, PsA L skin and PsA NL skin. Results are shown as median IOD/mm^2^ and IQR of epidermis and dermis of HD and PsA patients. Per subject the median expression level of 18 high-power fields is used for analysis. *p* values given in the graphs are calculated from a post Dunn’s test. E, epidermis; D, dermis, IL, interleukin; HD, healthy donor; PsA, psoriatic arthritis; L; lesional, NL; nonlesional, IOD; integrated optical density, IQR; interquartile range.

**Figure 4:**
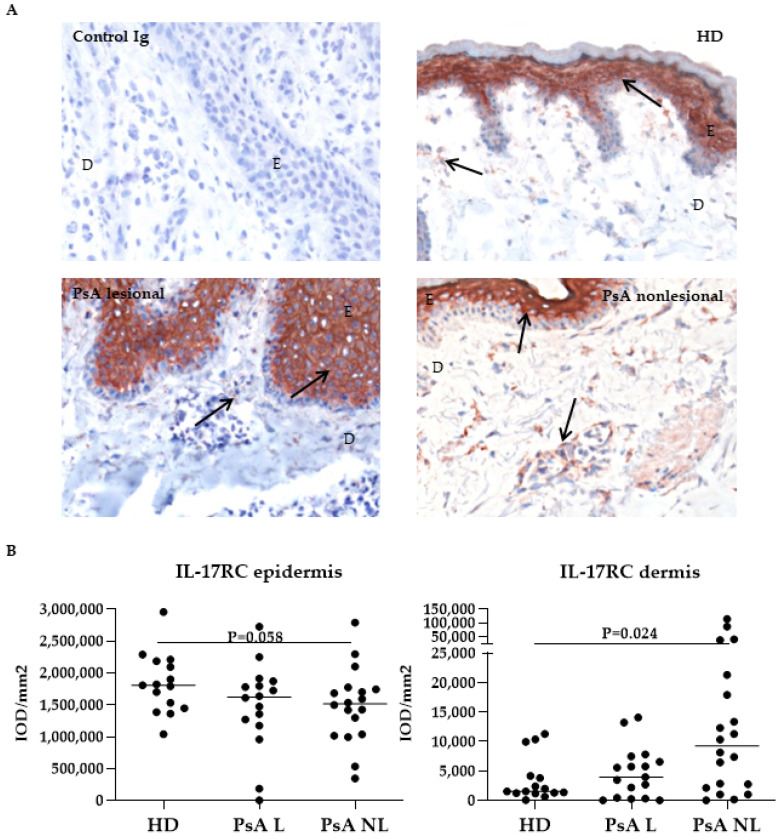
Expression of IL-17RC in psoriatic skin was higher in the dermis of nonlesional psoriatic skin compared to healthy donor skin. (**A**) Representative immunohistochemical staining of baseline IL-17RC expression (arrows) in skin from HD and PsA patients. Original magnification 200 ×. (**B**) Quantification of IL-17RC (Kruskal-Wallis test, epidermis *p* = 0.13, dermis *p* = 0.03) expression in HD, PsA L skin and PsA NL skin. Results are shown as median IOD/mm^2^ and IQR of epidermis and dermis of HD and PsA patients. Per subject the median expression level of 18 high-power fields is used for analysis. *p* values given in the graphs are calculated from a post Dunn’s test. E, epidermis; D, dermis, IL, interleukin; HD, healthy donor; PsA, psoriatic arthritis; L; lesional, NL; nonlesional, IOD; integrated optical density, IQR; interquartile range.

**Figure 5:**
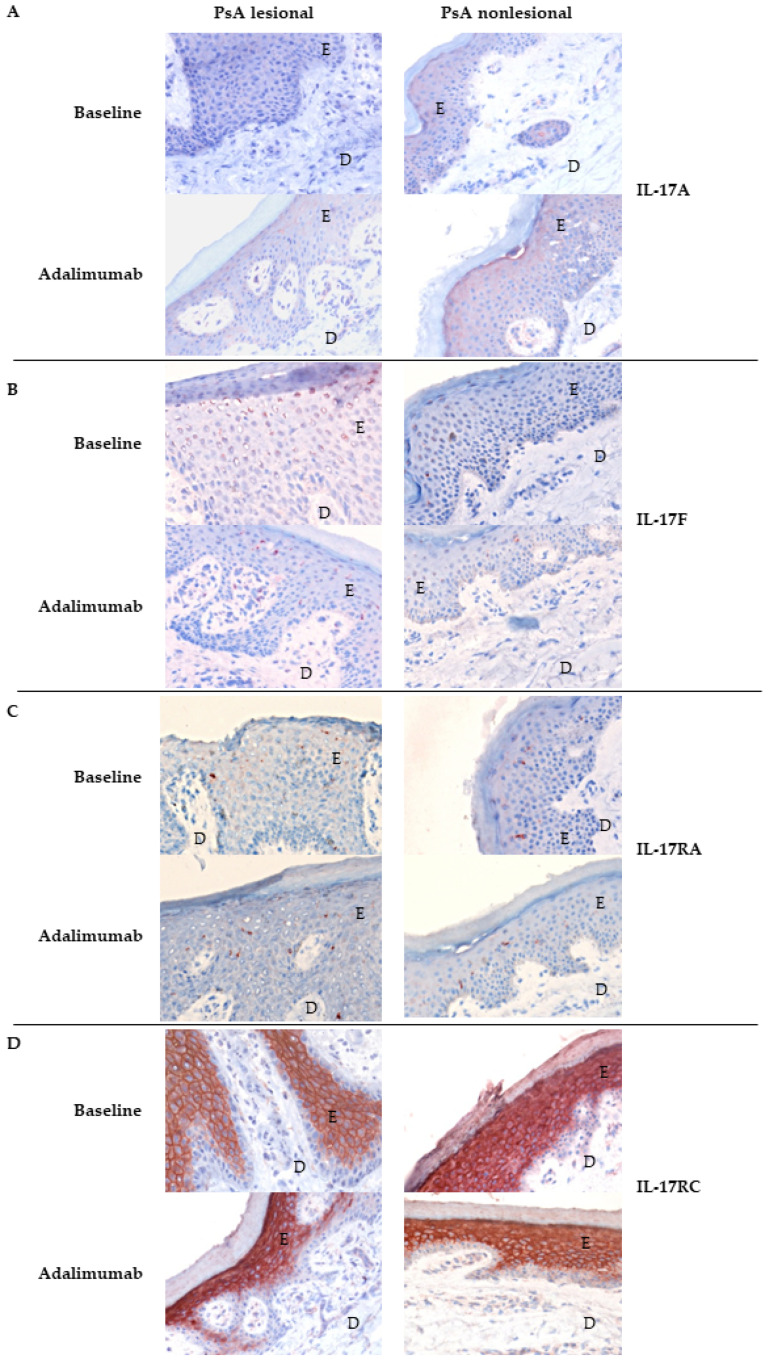
Representation of expression of IL-17A, IL-17F, IL-17RA and IL-17RC in psoriatic skin of one PsA patient before and after adalimumab treatment. Expression was not altered after 4 weeks of adalimumab. Representative immunohistochemical staining of IL-17A (**A**), IL-17F (**B**), IL-17RA (**C**) and IL-17RC (**D**) expression in skin of one PsA patient before (baseline) and after treatment with adalimumab. Original magnification 200 ×. E; epidermis, D; dermis, IL, interleukin; PsA, psoriatic arthritis.

In the lesional PsA skin, we observed typical psoriasis features, such as parakeratotic hyperkeratosis, acanthosis, perivascular lymphocyte infiltration and elongated rete ridges (Figure 1A, Figure 2A, Figure 3A, Figure 4A and Figure 5).

IL-17A expression showed a highly variable, diffuse and cytoplasmatic pattern (Figure 1A, arrows) in the epidermis, as well as in the dermis. Surprisingly, the levels of IL-17A were significantly lower in the epidermis and dermis of nonlesional PsA skin compared to HD skin (Figure 1B, *p* = 0.017 and *p* = 0.045, respectively). No significant differences were observed in the expression of IL-17A between lesional and nonlesional PsA skin (both in the epidermis and dermis). 

The expression of IL-17F showed a more cellular staining pattern (Figure 2A). In contrast to IL-17A, IL-17F was more expressed in the dermis of PsA lesional skin compared to HD skin (Figure 2B, *p* = 0.0002). No other significant differences were observed in the expression of IL-17F between lesional and nonlesional PsA skin (both in the epidermis and dermis), but levels were highly variable between patients.

IL-17RA was frequently expressed to varying degrees, mainly through the epidermis and, to a lesser extent, in the dermis (Figure 3A). The expression of IL-17RA was significantly reduced in the epidermis of lesional PsA skin compared to HD skin (Figure 3B, *p* = 0.007), while no differences were observed in the dermis. 

IL-17RC was abundantly expressed in the epidermis, but it was also found in dispersed cells throughout the dermis (Figure 4A). The expression of IL-17RC was significantly higher in the dermis of nonlesional PsA skin compared to HD skin (Figure 4B, right graph, *p* = 0.024), while IL-17RC expression levels were not significantly different in the epidermis.

### 3.2. Expression of IL-17A, IL-17F and Their Receptors in Skin of Psoriatic Arthritis Patients Treated with Placebo Compared to Adalimumab 

Available paired pre- and post- treatment skin and synovium tissue samples were used to quantify differences in the expression levels of each marker for each patient (in IOD/mm^2^) before and after treatment [29,30]. For each endpoint, ANCOVA was applied to correct for baseline differences between the placebo- and adalimumab-treated groups, and to compare the effect of adalimumab versus placebo after four weeks of treatment on IL-17 levels in skin and synovium. After 4 weeks of treatment, there was only a significant difference between the change in IL-17RA expression in the lesional dermis of the adalimumab group compared the lesional dermis of the placebo group (ANCOVA *p* = 0.01). This was not found for the other nonlesional and lesional IL-17RA expression levels in the epidermis and dermis. Of interest was the observation that the median expression of IL-17RA in the epidermis of the adalimumab group at baseline was 0 IOD/mm^2^. There were no significant differences after ANCOVA was applied for IL-17A (Table 2), IL-17F and IL-17RC alteration levels between placebo- and adalimumab-treated groups in the epidermis and dermis (*p* > 0.05). Overall, no clear effect was observed in IL-17 expression in skin upon adalimumab treatment. This is shown in Figure 5, which illustrates immunohistochemistry staining in skin tissues of one PsA patient before and after therapy with adalimumab. 

**Figure 6:**
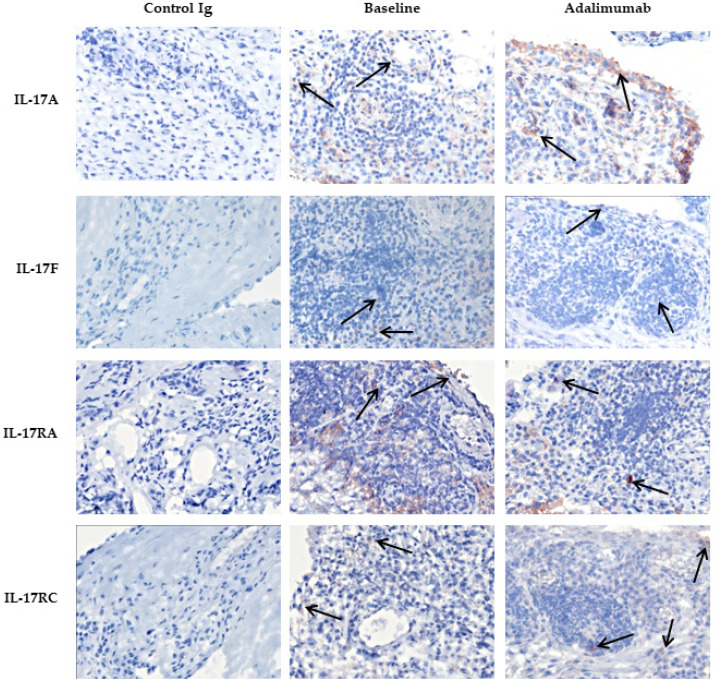
Representation of expression of IL-17A, IL-17F, IL-17RA and IL-17RC (arrows) in synovial tissue of one PsA patient before and after adalimumab treatment. Expression was not altered after 4 weeks of adalimumab. Representative immunohistochemical staining of IL-17A, IL-17F, IL-17RA and IL-17RC expression in synovial tissue of one PsA patient before (baseline) and after treatment with adalimumab. Original magnification 200 ×. IL, interleukin; PsA, psoriatic arthritis.

IL-17A and IL-17F expression were found both in the intimal lining layer and sublining of the synovium, and IL-17RA and IL-17RC were ubiquitously expressed (Figure 6).

Adalimumab treatment did not affect the expression of IL-17A, IL-17F, IL17RA or IL-17RC in PsA synovial tissue compared to the tissue of the placebo group, as all ANCOVA *p*-values were above 0.05 (Table 3). This was also illustrated by the immunohistochemistry of one PsA patient treated with adalimumab; see Figure 6. 

Overall, we found no significant positive correlation between IL-17 tissue levels and clinically relevant measurements such as C-reactive protein (CRP), erythrocyte sedimentation rate (ESR), PASI and DAS28 score.

## 4. Discussion

This is the first study to address the protein levels of IL-17A, IL-17F and their receptors in the skin of PsA patients with mild psoriasis (mean PASI score < 10) in comparison with HD skin. Additionally, it evaluated the early effects of adalimumab treatment on the expression of these molecules in both nonlesional and lesional skin and paired inflamed synovium samples of PsA patients. 

Overall, the histological expression levels of IL-17A, IL-17F, IL-17RA and IL-17RC in skin were highly variable among the PsA patients in our cohort. IL-17F levels were increased in the lesional dermis of PsA patients compared to HD skin, while IL-17A levels were not increased. This is in line with previous reports that found a higher IL-17F to IL-17A ratio on gene expression level in psoriatic skin [6]. In our cohort, the overall levels of IL-17A and IL-17F appeared low, as other studies have observed relatively high expression levels of these genes in psoriatic lesional skin compared to nonlesional psoriatic skin [6,35]. We found a lower level of IL-17RA in lesional psoriatic skin compared to HD skin and a higher expression of IL-17RC in the dermal nonlesional PsA skin compared to HD, which is not in line with earlier findings of increased gene expression levels of IL-17RA [35]. Possibly, the lower levels of IL-17A and IL-17RC observed in our study can be explained by the mild skin phenotype in our patients, as the median PASI in our study was 5.80, and only two patients had a PASI score just above 10, while other psoriatic skin studies reported PASI scores higher than 10 [35,36,37]. In addition, gene expression levels of total skin tissue biopsies may not correlate with protein levels quantified for either epidermis or dermis, especially for secreted cytokines, which are overall more challenging to quantify using immunohistochemistry [38]. Furthermore, we measured the IL-17 levels in IOD/mm^2^, which reflects IL-17 levels per square millimeter tissue but does not reflect the total presence of IL-17 protein levels in skin lesions, which often contain thickening of the epidermal layer, and thus, potentially harbor higher total IOD scores.

Several mechanisms for indirect downregulation of IL-17 levels by TNF blockade have been proposed. It was found in the skin lesions of psoriasis patients that TNF blockade with etanercept prevented TNF-mediated activation of myeloid dendritic cells, resulting in decreased IL-23 driven T-cell activation and a subsequent decrease in IL-17 levels in the skin [13]. Another study of psoriasis patients showed that the downregulation of IL-17 was caused by a decrease of IL-17R gene expression in keratinocytes after etanercept treatment [39]. In this study, we were unable to confirm the downregulation of either IL-17A or IL-17F. This may be explained by possible differences in molecular responses to the various TNF blockers, or by the relatively low IL-17 tissue levels observed at baseline in our study, which makes it more difficult to detect changes.

After four weeks of adalimumab treatment, we showed that there was no decrease in protein levels of IL-17A and IL-17F or their receptors in synovium and skin in our cohort of PsA patients with mild psoriasis. This was also not found in the skin and synovium samples from patients with significant improvements in the PASI or DAS28. In another study with psoriasis patients, IL-17 gene expression levels in the skin were decreased after 14 days of treatment with etanercept [39], but this study reported a much higher median body surface area (BSA) score for psoriasis, and thus, consisted of patients with more severe psoriasis. As the IL-17 protein levels detected in the skin of our PsA cohort were not very high, it may have been more difficult to observe a clear downregulation upon treatment. Although we observed a significant increase for IL-17RA in the epidermis of lesional skin of PsA patients after adalimumab treatment, the increase was relatively low, and significance was lost after adjustment for multiple comparisons. 

Though placebo-controlled, this study only included a limited number of patients, which makes it possible that we have missed small effects on cytokine expression. 

Initially, this study was designed to investigate the early effects of adalimumab on inflammation in synovium and skin. As the inclusion criteria were based on disease activity, swollen and tender joint counts, rather than the current extent of skin involvement, we included patients with low PASI scores and accordingly found smaller changes in PASI score upon adalimumab treatment compared to other cutaneous psoriasis trials [40]. 

Although there was no significant clinical improvement in the skin, a significant decrease in cellular infiltrate and DAS28 improvement was observed after four weeks of adalimumab [29,30]. All together this implies that in this cohort the clinical and cellular improvement after four weeks of treatment with adalimumab was probably independent of IL-17A, IL17-F, IL-17RA and IL-17RC levels.

Due to sample collection only after four weeks of adalimumab treatment, it is not excluded that IL-17 cytokine and receptor modulation is delayed and only effective after a longer period of treatment. This is stressed by the fact that the clinical efficacy of TNF-α blockade treatment with adalimumab starts already after 2 weeks, but is optimal after 12 weeks in terms of joint scores and 16 weeks in terms of skin scores [41]. Our study results are in line with two studies that observed no decrease in IL-17 blood levels in spondyloarthritis patients treated with TNF-inhibitors for 2 years [42] and 6 months [43]. The studies that evaluated IL-17 levels in blood samples have to be interpreted with caution as it is questionable whether these levels align with the effects on IL-17 levels observed target tissue like synovium and psoriatic skin. 

Of interest, another study found also no alterations in TNF mRNA levels in synovial tissues of PsA patients who were treated for 12 weeks with secukinumab, a monoclonal anti-IL17A antibody [44]. These and our findings suggest that the reduced synovial and skin inflammation observed in PsA patients after blocking of IL-17A is independent of changes in TNF levels, and vice versa, blocking of TNF is independent of changes in IL-17A levels. 

Our findings are of significance as the overall absence in downregulation of synovial and skin IL-17 protein levels upon TNF blockade supports the rationale that treatment of PsA patients with a combination of TNF and IL-17 blockers might be more beneficial, although the current study cannot exclude that changes in IL-17 levels might occur after a longer treatment duration. So far bispecific biologicals did not show an additional clinical effect compared to monotherapy with TNF blockade [45]. To the best of our knowledge, this is the first study documenting the effects of TNF-blockade on protein levels of IL-17 cytokines and receptors in paired skin and synovium samples of PsA patients. 

## 5. Conclusions

Altogether, our study shows that after four weeks of adalimumab treatment in PsA patients with mild skin involvement, TNF-blockade did not downregulate IL-17 cytokine and receptor protein levels in synovium and skin, despite improvement of the cellular infiltrate in both skin and synovium as well as clinical improvement, as measured by DAS28 score [29,30]. 

## Figures and Tables

**Table 1:** Demographic and clinical characteristics at baseline of the 24 patients with psoriatic arthritis.

	Adalimumab (*n* = 12)		Placebo (*n* = 12)	
Sex, female/male (*n*)	9/3		6/6	
Age, years	42.8	(21–61)	47.2	(25–78)
Patients on MTX, number (%)	7	(58%)	5	(42)
MTX dose, mg/week	18.2	(10–25)	19	(15–25)
Duration of psoriasis, years	8.8	(0.1–27.7)	20.8	(1.9–53.2)
Duration of PsA, years	5.5	(0.4–14.1)	8.4	(1.9–18.2)
DAS28 score	4.67	(3.0–5.78)	5.07	(2.21–6.83)
VAS disease activity (0–100 mm)	73	(45–94)	62.8	(18–92)
VAS pain (0–100 mm)	72.8	(55–91)	67.4	(11–89)
CRP, mg/liter	19.9	(2.3–81.6)	9.9	(1.3–26.7)
ESR mm/h	24.2	(4–66)	22.4	(3–66)
PASI	5.89	(0–14.0)	4.72	(0–7.0)

All values are mean (range) except where indicated otherwise. MTX, methotrexate; PsA, psoriatic arthritis; DAS, disease activity score; VAS, visual analogue scale (100 mm); CRP, C-reactive protein; ESR, erythrocyte sedimentation rate, PASI, psoriasis area severity index.

**Table 2:** Median IOD/mm^2^ values of IL-17 expression in lesional and nonlesional skin before and after treatment with adalimumab compared to placebo.

Skin Tissue		Adalimumab (*n* = 10)			Placebo (*n* = 11)			ANCOVA *p* Value
		IOD/mm^2^ Baseline		IOD/mm^2^ Change upon Treatment	IOD/mm^2^ Baseline		IOD/mm^2^ Change upon Treatment	
IL-17A	epidermis dermis	130,024 7261	(1421–406,897) (62–12,426)	1621 43	32,938 687	(10,501–75,100) (110–11,503)	2351 440	0.818 0.990
IL-17F	epidermis dermis	8127 13,436	(4276–20,978) (2979–25,674)	1583 9257	10,155 9591	(6351–30,021) (7997–29,477)	−6139 1379	0.945 0.812
IL-17RA	epidermis dermis	942 0	(0–3695) (0–42)	893 447	37 0	(0–4439) (0–0)	0 1	0.010 0.215
IL-17RC	epidermis dermis	1,473,790 5762	(726,625–1,679,188) (389–7648)	293,761 −3496	1,824,572 2679	(1,118,472–2,115,390) (247–5577)	26,775 1270	0.237 0.332
IL-17A	epidermis dermis	21,892 3297	(1015–20,420) (385–11,211)	323 189	35,249 1719	(5714–91,372) (461–7907)	3513 2436	0.945 0.083
IL-17F	epidermis dermis	1646 893	(0–1255) (0–44)	415 969	10,093 5960	(365–39,274) (970–22,719)	2372 644	0.760 0.914
IL-17RA	epidermis dermis	129 0	(773,173–1,689,893) (2378–25,450)	0 0	0 0	(0–1399) (0–30)	321 0	0.194 0.454
IL-17RC	epidermis dermis	1,496,622 10,226	(1,421–406,897) (62–12,426)	48,011 −3942	1,508,035 7371	(1,100,136–2,156,572) (1554–14,607)	213,162 −1483	0.339 0.375

IL-17A, IL-17F, IL-17RA and IL-17RC IOD/mm^2^ are expressed as median (IQR). Change upon treatment is expressed as median. A positive or negative value for change represents an increase or decrease in IOD, respectively. ANCOVA was applied to correct for baseline imbalance between placebo and adalimumab treatment groups. IOD; Integrated optical density, IQR; interquartile range.

**Table 3:** Median IOD/mm^2^ values of IL-17 expression in synovial tissue before and after treatment with adalimumab compared to placebo.

Synovial Tissue	Adalimumab (*n* = 11)				Placebo (*n* = 10)		ANCOVA *p* Value
	IOD/mm^2^ Baseline		IOD/mm^2^ Change upon Treatment		IOD/mm^2^ Baseline	IOD/mm^2^ Change upon Treatment	
IL-17A	7322	(1464–11,314)	12,211	2082	(228–17,070)	1581	0.333
IL-17F	19,297	(795–43,043)	4509	9146	(1015–21,304)	5495	0.811
IL-17RA	18,562	(2596–107,978)	−3676	682	(470–37,101)	2337	0.451
IL-17RC	3330	(1337–14,102)	283	3934	(800–5249)	230	0.385

IL-17A, IL-17F, IL-17RA and IL-17RC IOD/mm^2^ are expressed as median (IQR). Change upon treatment is expressed as median. A positive or negative value for change represents an increase or decrease in IOD, respectively. ANCOVA was applied to correct for baseline imbalance between placebo and adalimumab treatment groups. IOD; Integrated optical density, IQR; interquartile range.

## Data Availability

The data presented in this study are available on request from the corresponding author. The data are currently not publicly available.
